# Influence of Oxygen Diffusion on Thermal Ageing of Cross-Linked Polyethylene Cable Insulation

**DOI:** 10.3390/ma13092056

**Published:** 2020-04-29

**Authors:** Yuanyuan Zhang, Zongke Hou, Kangning Wu, Shihang Wang, Jianying Li, Shengtao Li

**Affiliations:** State Key Laboratory of Electrical Insulation and Power Equipment, Xi’an Jiaotong University, Xi’an 710049, China

**Keywords:** XLPE, thermal ageing, oxygen diffusion, non-uniform

## Abstract

Thermal ageing of cross-linked polyethylene (XLPE) cable insulation is an important issue threatening the safe operation of power cables. In this paper, thermal ageing of XLPE was carried out at 160 °C in air for 240 h. The influence of oxygen diffusion on thermal ageing of XLPE was investigated by Ultraviolet–visible spectrophotometer (UV–Vis), tensile testing, and Fourier transformed infrared spectroscopy (FTIR). It was observed that the degradation degree not only depended on ageing time but also on sample positions. The thermally aged samples were more oxidized in the surface region, presented a darker color, more carbon atoms appeared in the conjugate cluster, had smaller elongation at break and tensile strength, and a larger carbonyl index. As ageing time increased, the non-uniform oxidation of the XLPE samples became more prominent. The degree of non-uniform oxidation caused by oxygen diffusion was quantitatively studied by first order oxidation kinetic. The calculated results demonstrated that carbonyl index measured by FTIR was more sensitive to non-uniform oxidation with a shape parameter in the range of 1–2. The result shown in this paper is helpful for interpreting and predicting the non-uniform ageing behavior of high voltage XLPE cables.

## 1. Introduction 

Cross-linked polyethylene (XLPE) has been extensively used as insulating material for high voltage power transmission and distribution cables because of its excellent mechanical, thermal, and electrical properties [1,2,3]. The performance of XLPE is inevitably affected by various stresses during operation [4]. Thermal ageing is considered as one of the major factors causing the modification of structure and deterioration of properties [5,6]. It is of great significance to study thermal ageing behavior of XLPE in order to guarantee the safety and reliability of power cables.

A considerable amount of literature has been published about the thermal ageing of XLPE. These studies were mainly carried out by performing accelerated thermal ageing experiments in air to XLPE cable or cable peelings [7,8]. The changes of properties and molecular structures were characterized by a variety of techniques [9,10]. The elongation at break which is a key parameter for XLPE cable insulation was found to decrease as thermal ageing proceeded [11]. The electrical breakdown strength decreased with the ageing degree while the conductivity and dielectric loss increased [12,13]. Thermal properties of XLPE cable insulation can be studied by differential scanning calorimetry (DSC) and thermogravimetric analysis (TGA). It was reported that melting peak temperature, melting enthalpy, and crystallinity decreased with ageing time at high temperatures (120 and 140 °C) because of the chain scission. When aged under low temperatures (80 and 100 °C), the crystallinity of XLPE increased due to recrystallization from freed segments [14]. The TGA results showed that thermal stability of XLPE decreased after ageing, leading to the increase in the propagation rate of the electrical trees [15]. Fourier transformed infrared spectroscopy (FTIR) studies showed that the oxidation products increased with the oxidation degree [16]. The thermal ageing of XLPE has been accepted as a process of oxidation chain reaction essentially, involving a participation of free radicals and peroxides [17]. These studies have significantly contributed to the understanding and prediction of the XLPE thermal ageing behavior.

It is worthwhile to notice that oxygen diffusion is a critical factor for polymeric materials when aged under oxidative conditions [18,19]. The oxygen dissolved in the material is consumed by oxidation reactions under a thermal ageing process. At the same time, oxygen molecules in air diffuse into the material and continue to participate in the oxidation reactions. When the oxygen consumption rate is larger than the diffusion rate, the oxygen in the sample cannot be supplied in time. The reduced oxygen concentration in the sample finally leads to a decrease of oxidation degree. Therefore, non-uniform ageing is observed with a higher oxidation level in the surface than that in the interior.

The non-uniform oxidation of XLPE has been reported in several studies. The research results showed that the density and carbonyl index increased dramatically in the surface after radiation at a large dose rate while little change was observed in the interior region [20,21]. It was found that the fast oxygen consumption rate in the surface resulted from high a dose rate. The effect of oxygen diffusion needs to be eliminated or carefully interpreted since it would complicate or even prevent the extrapolation of changes in material properties from the accelerated condition to the actual operation condition [22,23]. Although the influence of thermal ageing on XLPE has been well studied, the role of oxygen diffusion on the ageing of high voltage XLPE cable insulation has not been considered. For thin XLPE films, oxygen consumed in the sample can be quickly supplied by the diffusion process. The oxidation is uniform and the influence of oxygen diffusion is often unimportant. However, oxygen diffusion cannot be neglected because of the large thickness of high voltage XLPE cable.

The aim of this research is to quantitatively investigate the influence of oxygen diffusion on thermal ageing of thick XLPE cable insulation, which would increase the knowledge about ageing of high voltage cable insulation. In this paper, a 110 kV XLPE cable insulation was aged at 160 °C in air for 240 h. The degradation along radial direction of XLPE during the thermal ageing process was studied by Ultraviolet–visible spectrophotometer, tensile testing, and FTIR. The first order oxidation model was introduced for a better understanding of the non-uniform ageing behavior of thick XLPE cable insulation.

## 2. Materials and Methods

### 2.1. Sample Preparation

The sample used in this study was a new commercial 110 kV ac XLPE cable (Qingdao Hanhe Cable Co., Ltd., Qingdao, China). The cylinder XLPE insulation samples were prepared by peeling off the sheath and outer semiconductor shield layer of the cable. The thickness of the remaining XLPE insulation sample was about 13 mm. Accelerated thermal ageing was performed by putting the cylinder XLPE samples in an air-forced oven at 160 °C. After exposure for 48, 96, 144, 192, and 240 h, the samples were taken out and cut spirally into long tapes for characterization.

**Figure 1:**
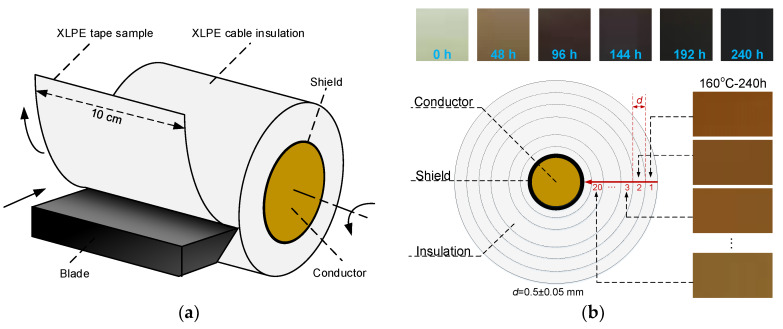
(**a**) The schematic diagram of cutting method; (**b**) the distribution of cross-linked polyethylene (XLPE) at different positions. The top is the surface color of the cylinder samples and the right is the color of the sheet samples.

The schematic diagram of the sample cutting method is illustrated in Figure 1a. The cylinder XLPE sample was installed in a rotary machine and cut by a lathe whose blade was moving forward. The cutting process was controlled by a numerical control machine. The thickness of the tape samples was achieved by adjusting the rotation speed of the cable and the moving speed of the blade. A long tape with a width of 10 cm and a thickness of 0.5 ± 0.05 mm was obtained. Then, the tape was divided according to radial intervals of about 0.5 mm from the outer surface to the inner conductor. The samples were denoted as 1, 2, 3, …, 20 as shown in Figure 1b. The relative positions of sheet samples were normalized into 0–1.

This method has been frequently applied in preparation of a cable insulation sample [24,25]. When the blade was close to the copper conductor, there was a risk of collision between conductor and blade. Therefore, few insulation residue near the conductor was left after processing, leading to a lower number of sections than 26. We chose 20 sections for all XLPE samples to study the dependence of thermal ageing on sample positions.

### 2.2. Characterization

The optical property of XLPE was characterized by color change and a Ultraviolet–visible spectrophotometer (UV–Vis, Shimadzu UV-3600, Kyoto, Japan). The absorption spectra in the wavelength range of 200–700 nm were recorded by mounting the sample in an integrating sphere. The reference curve was kept as air. The measurement was repeated twice for each sample. The UV–Vis absorption curves from the second measurement were presented in this paper. Calculation data points are the average value of two pieces.

**Figure 2:**
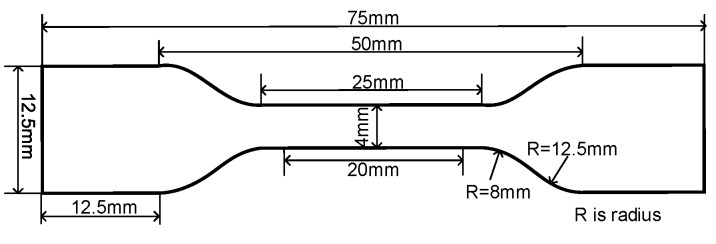
Schematic diagram of XLPE dumbbell sample for the tensile test.

Changes in mechanical property of the samples were measured by a tensile test with a universal testing machine (5kNCMT-4503, MTS Industrial Systems Co., Ltd., Shanghai, China). The samples were cut into a dumbbell shape with a cut-off knife and punching machine. The specific size of the dumbbell shape sample is shown in Figure 2 [26]. The extension rate was 100 mm/min. The elongation at break and tensile strength data points were the average value of five pieces.

The ageing products studies were carried out by Fourier transformed infrared spectroscopy (FTIR, Nicolet iN10, Madison, WI, USA) ranging from 4000 to 500 cm^−1^ in transmission mode. FTIR spectra were obtained using scan summations of 32 and a resolution of 4 cm^−1^. For each XLPE sample, two pieces were measured. The FTIR spectra from the second measurement were analyzed in this research. Relative data points are the average value of two pieces.

## 3. Results

### 3.1. Influence of Thermal Ageing on Optical Properties

The visual change is the first impression of degradation when a polymer is exposed to thermal ageing. As presented in the top of Figure 1b, the surface color of the entire cylinder XLPE sample (thick insulation with conductor) changes from white to yellow after ageing for 48 h and it gets darker with increasing ageing time. For each aged XLPE sample, the discoloration is also observed in radial direction when they were cut into sheets with a thickness of 0.5 mm. The sheet sample aged after 240 h presents more red in the surface region while the interior region is yellow as shown on the right side of Figure 1b. The surface color of the cylinder sample seen in the top of Figure 1b is the compositive expression of all sections while the color shown in the right belongs to each section. Therefore, the color of cylinder sample is black after 240 h while the color of section 1 appears red. The color of the XLPE samples aged for 48, 96, 144 and 192 h in radial direction also varies gradually, which is similar with the sample aged for 240 h, thus not presented here.

**Figure 3:**
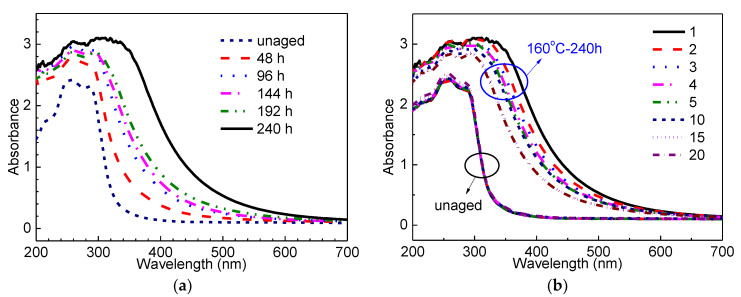
(**a**) UV–Vis absorption spectra of the No.1 sample as a function of ageing time; (**b**) UV–Vis absorption spectra of unaged and aged for 240 h XLPE samples at different positions.

The discoloration of XLPE with ageing time and sample position was further studied by UV–Vis. Figure 3a shows the absorption spectra of the No.1 XLPE sample with different ageing time. It is clear that the absorption edge is shifted to a longer wavelength with increasing ageing time. Figure 3b compares the absorption spectra of unaged and aged for 240 h XLPE samples at different positions. The absorption curves of unaged samples at different positions are nearly identical. On the contrary, the absorption behavior of thermally degraded samples are quite dependent on the sample positions. As can be seen in Figure 3b, the No.1 section of the thermally aged sample has largest absorbance at each wavelength and absorption edge. It obviously indicates that the shift of the absorption edge towards a longer wavelength is consistent with the sample discoloration.

The shift of the absorption edge towards longer wavelength direction is generally attributed to the formation of radicals, unsaturated bands, and conjugated bands [27]. As shown in Figure 3a,b, the absorption maximum at 215 nm and 256 nm come from the electronic vibration of –(CH=CH)_2_– and –(CH=CH)_3_–. The absorption band located at 285 nm is the characteristic of transition in unsaturated ketones (–C=C-C=O–). The formation of conjugated –CH=CH– bonds and unsaturated ketones (–C=C-C=O–) upon thermal ageing could be result of the coupling of –C=C– and –C=O–. The –C=C– and –C=O– were generated when radicals deactivate one another in pairs [28].

It was reported that the average number of carbon atoms per conjugate cluster (*N*) could be estimated from UV–Vis spectra [29]. The number of carbon atoms in a conjugate cluster means the number of carbon atoms participating in the conjugation effect. For example, *N* is 4 for the conjugate cluster 1,3-Butadiene (CH_2_=CH–CH=CH_2_). For polyethylene, *N* is an average number and can be calculated by Equation (1) [30].

(1) $$N=2\beta \pi /E_{g}$$

where *2βπ* is the band gap energy of a pair of adjacent *π* sites. The value of *β* is 2.9 eV as it is associated with π–π^*^ optical transitions in –C=C– structure. *E_g_* is the band gap energy of XLPE, which can be calculated by Equation (2) [31]

(2) $$E_{g}=hc/\lambda_{g}$$

where *h* = 6.626 × 10^−34^ *J·s* is the plank constant, *c* = 3.0 × 10^8^ m/s is the velocity of light, and *λ*_g_ is the absorption edge obtained from UV–Vis spectra.

**Figure 4:**
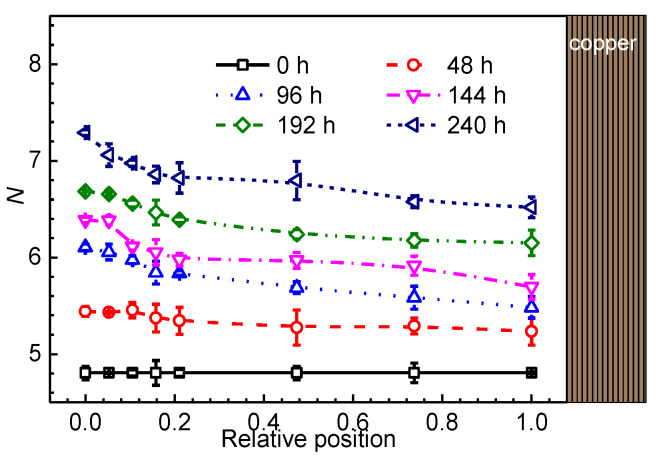
Profile of *N* for different XLPE samples.

The variation of *N* as a function of sample relative position during thermal ageing is presented in Figure 4. It can be found that *N* increases after thermal ageing and the value increases from about 5 for the unaged sample to 7 for the sample aged for 240 h. It is notable that the value is larger near the surface region than that in the interior region. The increase of *N* during thermal ageing contributes to the discoloration of the XLPE sample from white transparent to yellow opaque. These results clearly demonstrate that the degradation in optical property is non-uniform and the surface region is more affected by the thermal ageing condition.

### 3.2. Influence of Thermal Ageing on Mechanical Properties

**Figure 5:**
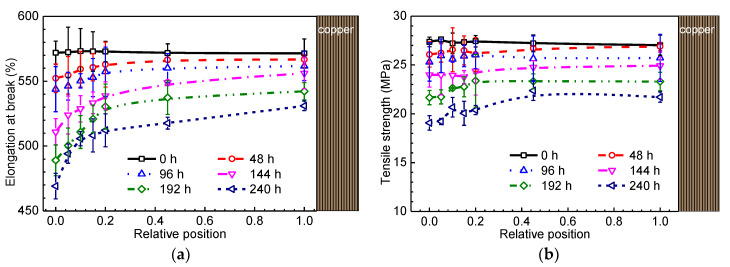
(**a**) Profile of elongation at break for different XLPE samples; (**b**) profile of tensile strength for different XLPE samples.

Tensile testing has been frequently employed to evaluate the degradation degree of polymeric insulating materials. Figure 5 shows the dependence of mechanical properties including elongation at break and tensile strength on ageing time and sample relative position.

As shown in Figure 5a,b, the elongation at break is about 570% and the tensile strength is about 28 Mpa for the unaged XLPE sample no matter where the sample position is. With the increase of ageing time, the elongation at break and tensile strength decrease gradually as frequently reported in the literature [11]. Furthermore, the decreases of elongation at break and tensile strength are dependent on the sample position. The dramatic reduction in elongation at break and tensile strength is observed in the surface region of the aged samples. Longer ageing time leads to greater difference in elongation at break and tensile strength between the surface and interior region. The drop of elongation at break and tensile strength indicates the reduction of molecular chain flexibility which arises from the rupture and oxidation of molecular chains. It was reported that the mechanical properties decreased with an increase in the thermal oxidation degree [32], which certifies the close relation between non-uniform degradation and oxygen diffusion when aged under 160 °C.

### 3.3. Influence of Thermal Ageing on Oxidation Products

**Figure 6:**
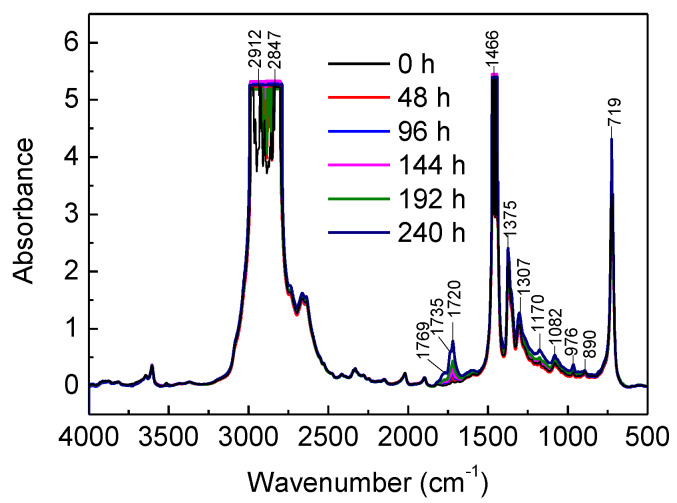
FTIR spectra of the No.1 sample as a function of ageing time in the range of 4000–500 cm^−1^.

FTIR spectroscopy is acknowledged as a powerful tool to detect oxidation products by characterizing molecular rotation and vibration [33]. The FTIR spectra of the No.1 XLPE sample during thermal ageing are shown in Figure 6. The absorption peaks at 2912 cm^−1^ and 2847 cm^−1^ correspond to the asymmetric and symmetric stretching vibration of –CH_2_, respectively. The peaks at 1466 cm^−1^ and 719 cm^−1^ are from the wag and rocking vibration of –CH_2_, respectively. The decrease of the XLPE characteristic peaks (719, 1466, 2847, and 2912 cm^−1^) after ageing was attributed to the molecular chain scission [34]. In addition, an increase in absorbance located in the range of 1400–800 cm^−1^ can be observed. The absorption peaks at the 1307 and 1170 cm^−1^ regions are assigned to –C–O–C vibrations [35]. The peaks at 1082, 976, and 896 cm^−1^ are related to unsaturated C=C groups [27]. The increase of carbonyl groups is observed in the range of 1850–1650 cm^−1^. Since the carbonyl groups are closely related to the oxidation degree, the FTIR spectra in carbonyl regions were further analyzed.

**Figure 7:**
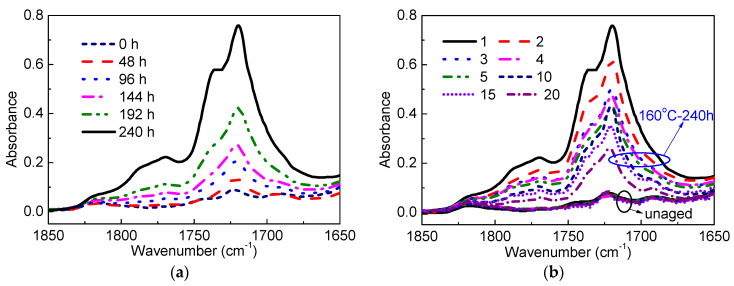
(**a**) FTIR spectra of the No.1 sample as a function of ageing time; (**b**) FTIR spectra of unaged and aged for 240 h XLPE samples at different positions.

Figure 7a depicts the spectra of sample No.1 in carbonyl stretching regions at different ageing times. It is observed that the absorbance of carbonyl groups increases dramatically as thermal ageing progresses. Three absorption peaks are observed in the carbonyl regions. The 1720 cm^−1^ peak is assigned to the carbonyl in the ketone group. The carbonyl peak at 1735 cm^−1^ belongs to the ester. The stretch of C=O in γ-lactone appears at 1769 cm^−1^. The accumulation of three carbonyl groups with ageing time indicates the increase of the oxidation degree of the XLPE samples.

The FTIR spectra of XLPE samples located at different positions are shown in Figure 7b. The absorbance is quite small and minor differences are detected between each section of unaged samples. After ageing for 240 h at 160 °C, the spectra become position dependent from sample No.1 to No.20. Considerable non-uniformity in distribution of oxidation products is observed with deeper oxidation in the surface region than in the interior region.

**Figure 8:**
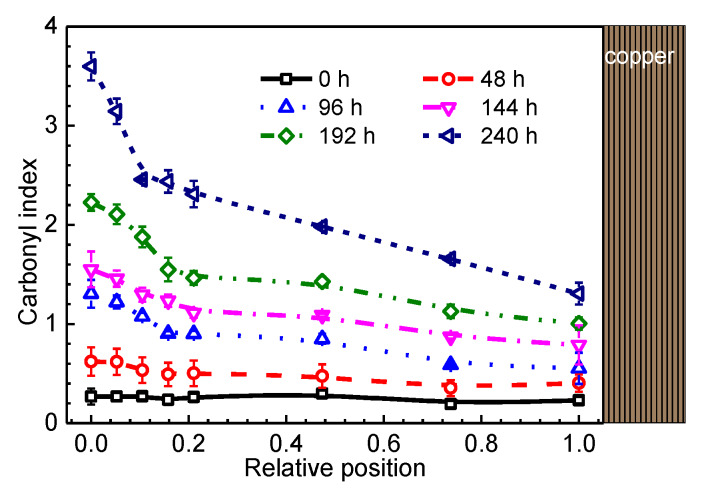
Profile of carbonyl index for different XLPE samples.

To describe the oxidation products quantitatively, carbonyl index is calculated by the ratio between the area of carbonyl band (1850 cm^−1^–1650 cm^−1^) and internal standard peak at 2010 cm^−1^ [7]. The dependence of the carbonyl index on the relative position of the sample is shown in Figure 8. As expected, the profile of the carbonyl index is not only time dependent but also position dependent. At the initial ageing stage, the carbonyl index profile is flat and then it evolves with ageing time to become highly non-uniform. The results in Figure 8 clearly indicate the non-uniform oxidation of XLPE cable insulation.

## 4. Discussion

Thermal oxidative ageing of polymeric materials has been an important subject for several decades. Great effort has been made by many researchers on the study of ageing characteristics. Based on the characterization of material structure and properties, the ageing mechanism was proposed to explain various experimental phenomena. It is widely accepted that the thermal oxidation process of XLPE can be described by an auto-oxidation scheme. During thermal ageing, the weak chemical C–H bonds in XLPE are broken, leading to a formation of free radicals R·. These radicals R· are instantly turned to peroxy radicals ROO· by oxygen. Then, ROO· can capture hydrogen atoms again from molecular chains generating hydro-peroxides ROOH. Once produced, ROOH can decompose to RO· and OH·, resulting in successive oxidation reactions. The termination of R·, RO·, and ROO· finally forms the inactive products such as carbonyl groups, as well as unsaturated and conjugated groups. Therefore, the chain scission and oxidation reactions result in the discoloration and decrease of elongation at break after thermal ageing.

On the basis of the above analysis, the non-uniform oxidation of XLPE is well understood and can be explained by the oxygen diffusion theory. For the thick XLPE sample, oxygen in air diffuses into the XLPE sample and participates in oxidation reactions with molecular chains. The large thickness means that a longer time is needed for oxygen to diffuse into the interior of the sample. Therefore, the oxygen consumed by oxidation reactions cannot be replenished in time, leading to a region in the interior with reduced oxygen concentration. The degree of non-uniform oxidation is closely dependent on oxygen concentration distribution in the sample according to the auto-oxidation scheme. For the purpose of quantifying the non-uniform oxidation behavior, the oxygen concentration (O_2_) in XLPE at different positions is calculated theoretically according to Fick’s second law [36]. The oxygen consumption rate satisfies Equation (3)

(3) $$\frac{{d(O}_{2})}{dt}=D\frac{d^{2}{(O}_{2})}{dx^{2}}-r$$

where *t* is time, *D* is oxygen diffusion coefficient, *x* is the distance to the surface, and *r* is the reaction rate of oxygen. To simplify the model, it is assumed that *r* is proportional to the oxygen concentration. In an equilibrium state, Equation (3) becomes Equation (4)

(4) $$D\frac{d^{2}{(O}_{2})}{dx^{2}}-k(O_{2})=0$$

where *k* is the rate constant. The solution of Equation (4) is shown in Equation (5)

(5) $$\frac{{{(O}_{2})}_{x}}{{{(O}_{2})}_{0}}=\frac{\cosh(\theta (x-l))}{\cosh(\theta l)}$$

where (O_2_)*_x_*/(O_2_)_0_ is the relative oxygen concentration at depth *x*, *l* is the thickness of the sample, and *θ* = (*k*/*D*)^0.5^ is called the shape parameter which determines the degree of non-uniform for oxygen concentration curve.

**Figure 9:**
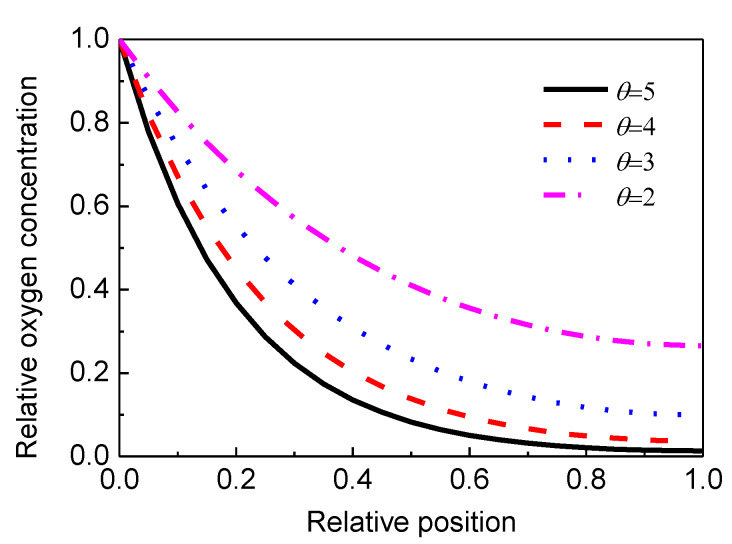
Computed profile of relative oxygen concentration with different.

Figure 9 plots the computed relative oxygen concentration profile with different values of *θ*. It is clearly apparent that the profile of relative oxygen concentration has a similar shape with the profile of *N*, elongation at break, and the carbonyl index as shown in the experimental results. Greater value of *θ* means steeper shape of the curve and larger degree of non-uniform oxidation. When the reaction rate constant *k* and oxygen diffusion coefficient *D* are experimentally accessible, *θ* can be obtained. It can be expected that *θ* becomes larger when XLPE is aged at higher temperature since a higher temperature gives larger *k*. Therefore, the accelerated thermal ageing temperature of the complete 110 kV XLPE cable insulation should be lower than 160 °C when uniform oxidation is required.

**Figure 10:**
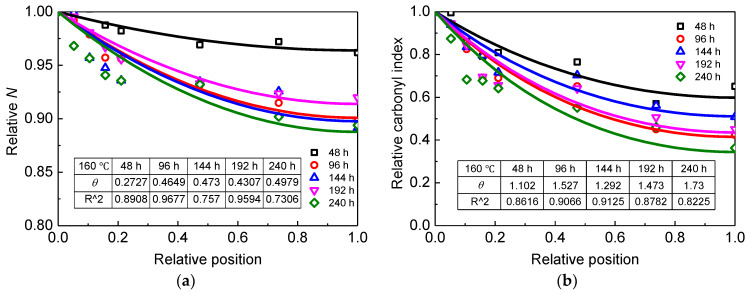
(**a**) Relative *N* of aged XLPE samples; (**b**) relative carbonyl index of aged XLPE samples. Dots are measured data points and lines are fitted results using Equation (5).

The conjugate cluster measured by UV–Vis and carbonyl groups measured by FTIR are closely related to the oxidation reactions, while the elongation at break would be influenced by other factors in addition to oxidation reactions. In this paper, it is reasonable to use *N* and the carbonyl index to determine the degree of non-uniform oxidation of XLPE aged at 160 °C. For simplicity, it is assumed that *N* and the carbonyl index are proportional to the oxygen concentration [36]. Therefore, the relative *N* and carbonyl index can be expressed by Equation (5). Consequently, the relative *N* and carbonyl index data points of aged XLPE were fitted by Equation (5) and presented as lines in Figure 10. The inset table in Figure 10 illustrates the calculated values of *θ* and goodness of fit R^2^.

It is observed that *θ* obtained from UV–Vis is in the range of 0.2–0.5, while *θ* is in the range of 1–2 from carbonyl index measured by FTIR. The common feature of the fitting result is that *θ* tends to increase with ageing time. The difference of *θ* may be attributed to the sensitivity of parameters to the oxidation degree. In other words, the carbonyl index may be more sensitive to oxidation and can be used to determine the degree of non-uniform oxidation of XLPE. The agreement between the theory and experiment is achieved since R^2^ is about 0.9. To date, the quantitative study of non-uniform oxidation for XLPE cable insulation has not been reported, whereas a similar study was noticed in the thermal ageing of polyamide 6. It was found that the shape parameter of polyamide 6 obtained from the carbonyl index (7.9 aged at 120 °C) was also larger than that from absorbance at 280 nm (5.1 aged at 120 °C) [37]. According to the expression of the shape parameter, the different values of the shape parameter between XLPE and polyamide 6 may be ascribed to the difference in the diffusion coefficient and reaction rate constant.

It can be concluded that the non-uniform oxidation of XLPE under thermal ageing can be described by first order oxidation kinetics. Oxygen diffusion is a critical factor for materials aged in accelerated conditions in an aerobic atmosphere, which often leads to spatially dependent degradation. In addition to the optical and mechanical properties, thermal properties would also be influenced by oxygen diffusion during thermal ageing. Further study is still needed to disclose a full picture of non-uniform oxidation of high voltage XLPE cable insulation. Our research results would be beneficial to understand and determine the characteristics of non-uniform degradation for high voltage XLPE cable insulation.

## 5. Conclusions

In this paper, the influence of oxygen diffusion on thermal ageing of high voltage XLPE cable insulation was investigated. Several conclusions were shown as follows:

(1) Significant non-uniform oxidation of XLPE was caused by oxygen diffusion during thermal ageing. The color, average number of carbon atoms per conjugate cluster, elongation at break, tensile strength, and carbonyl index of XLPE varied spatially along radial direction;
(2) The degradation of aged samples in the surface region was more severe than that in the interior region. As ageing time increased, the oxidation behavior transited from uniform to non-uniform;
(3) The degree of non-uniform degradation of XLPE can be calculated by first order oxidation kinetic. The carbonyl index measured from FTIR was more sensitive to non-uniform oxidation than the average number of carbon atoms per conjugate cluster from UV–Vis;
(4) The ageing temperature should be lower than 160 °C when uniform oxidation of 110 kV XLPE cable insulation was required.
